# Predictive Value of Bleb Vascularity after Mitomycin C Augmented Trabeculectomy

**DOI:** 10.3390/jcm9113501

**Published:** 2020-10-29

**Authors:** Aleksandra Wlaź, Anna Kuna, Agnieszka Wilkos-Kuc, Agnieszka Rozegnał-Madej, Tin Aung, Tomasz Żarnowski

**Affiliations:** 1 Department of Diagnostics and Microsurgery of Glaucoma, Medical University, Chmielna 1, 20-079 Lublin, Poland; ryan84@onet.eu (A.K.); agnieszkawilkos@wp.pl (A.W.-K.); madejaga@o2.pl (A.R.-M.); zarnowskit@poczta.onet.pl (T.Ż.); 2 Singapore Eye Research Institute & Singapore National Eye Centre, 11 Third Hospital Avenue, Singapore 168751, Singapore; aung_tin@yahoo.co.uk; 3 Yong Loo Lin School of Medicine, National University of Singapore, 10 Medical Drive, Singapore 117597, Singapore

**Keywords:** trabeculectomy, filtering bleb, glaucoma surgery, wound healing, vessel area

## Abstract

Background: To evaluate the relationship between bleb vascularity and surgical outcome one year after mitomycin C (MMC) augmented trabeculectomy. Methods: This was a prospective study of 51 eyes of 44 patients after MMC-augmented primary trabeculectomy with follow-up of 12 months. The total vessel area of a bleb was measured with ImageJ software on color photographs of the bleb on day 1 and 14, then months 1, 3, 6 and 12 after trabeculectomy. Blebs were classified clinically as successful (intraocular pressure (IOP) ≤ 18 mmHg and a >30% reduction in IOP without antiglaucoma medications or additional surgical interventions) or failed. Linear regression analysis was performed to determine the correlation of bleb vascularity with IOP and outcome. Results: At 1 year, 40 eyes (78.4%) were classified as successful and 11 eyes (21.6%) as failed. The mean bleb vascularity at 1, 3 and 12 months after surgery was significantly higher in failed blebs (16.31% vs. 13.01%, *p* = 0.005, 14.93% vs. 10.15%, *p* = 0.001, 8.99% vs. 6.37%, *p* = 0.011, respectively). There were no significant differences in mean bleb vascularity at 1 and 14 days postoperatively in successful and failed blebs. The results revealed a significant association between vessel area at 1 and 3 months after trabeculectomy with IOP at 6 months postoperatively (*p* = 0.005 and *p* = 0.009, respectively). Conclusions: In this prospective study, we demonstrated a strong relationship between bleb vascularity and the surgical outcomes of trabeculectomy. Vascularity of the filtering bleb during early postoperative period was not correlated with IOP or success of trabeculectomy at one year. Increased bleb vascularity 1, 3 and 12 months after trabeculectomy appears to predict surgical failure at 1 year after trabeculectomy.

## 1. Introduction

The success of glaucoma filtering surgery depends on constant shunting of aqueous humor from the anterior chamber to the subconjunctival space. Excessive subconjunctival scarring is the main reason for failure of this procedure.

Angiogenesis plays a crucial role in physiological wound healing and scar formation [1]. Increased vascularity of the filtering bleb during first several weeks after surgery may herald subconjunctival fibrosis and failure of the procedure. Research has showed that introduction of the antiangiogenic agents to the postoperative care may increase the success rate of filtering surgery [2,3].

One of the crucial prognostic factors of outcome of trabeculectomy surgery is the morphology of the filtering bleb. In the early postoperative period, evaluation of blebs is therefore essential for follow-up of glaucoma surgery. Picht and Grehn [4] reported that filtering blebs with fewer conjunctival and corkscrew vessels are more likely to have a better surgical outcome. Recently, Yin et al. [5] prospectively analyzed the correlation of the vessel area of the filtering bleb after trabeculectomy with surgical outcomes using optical coherence tomography angiography (OCT-A).

To date, several studies have described the use of slit lamp microscopy [4], ultrasound biomicroscopy (UBM) [6], in vivo confocal microscopy (IVCM) [7,8,9] and anterior segment optical coherence tomography (AS-OCT) [10,11] for investigating bleb morphology. Several clinical classification systems have long been used to assess the morphology of blebs, e.g., the Indiana Bleb Appearance Grading Scale (IBAGS) [12] and the Moorfields Bleb Grading System (MBGS) [13]. These methods subjectively evaluate characteristics of the filtering bleb such as the vessel area on slit-lamp examination. Usually, color photographs are obtained in order to enable more repeatable and standardized evaluation of blebs.

In this study, we prospectively evaluated bleb vascularity using color photographs and examined the correlation between vessel area and surgical outcome of trabeculectomy.

## 2. Materials and Methods

### 2.1. Trial Registration

ClinicalTrials.gov registration number NCT04552314.

### 2.2. Study Design

This was a prospective study of 51 eyes of 44 patients with medically uncontrolled glaucoma who underwent primary trabeculectomy with mitomycin C (MMC) in the Department of Diagnostics and Microsurgery of Glaucoma of Medical University of Lublin, Poland. The follow-up was 12 months and patients were enrolled between July 2014 and March 2015. The protocol of this study was approved by the Bioethics Committee of Medical University in Lublin (Lublin, Poland) and registered under the number KE-0254/203/2014. The aims of the study, the benefits and risks of the procedure were explained thoroughly to the patients and written informed consent was obtained from all participants in accordance with tenets of Declaration of Helsinki.

### 2.3. Inclusion and Exclusion Criteria

The inclusion criteria of the study were patients aged over 18 years with primary open angle glaucoma (POAG) or pseudoexfoliation glaucoma (PEXG) with medically uncontrolled intraocular pressure (IOP). Glaucoma was defined as the presence of typical glaucomatous optic neuropathy with characteristic visual field defects on automated perimetry confirmed by 30-2 SITA Standard Humphrey visual field analysis. Exclusion criteria were primary angle closure glaucoma (PACG), secondary glaucoma except PEXG, previous eye surgery or subjects with decreased vision due to reasons other than glaucoma (e.g., exudative age-related macular degeneration (AMD), proliferative diabetic retinopathy, inflammatory eye diseases). The eyes with subconjunctival hemorrhages on day 1 after trabeculectomy were also excluded.

### 2.4. Study Protocol

Before surgical intervention, all patients underwent a baseline examination which included assessment of best corrected visual acuity (BCVA) (LogMAR charts, CP-400 Frey SJ, Piaseczno, Poland), biomicroscopy with dilated evaluation of the fundus with a 78 D lens and measurement of IOP (Goldmann applanation tonometry) with number of ocular hypotensive medications. After surgical intervention, follow-up visits were arranged at 1 day, 1 and 2 weeks, 1, 3 and 6 months and 1 year after surgery. At each visit, all the above-mentioned examinations were performed. Postoperative complications and secondary procedures performed after trabeculectomy were recorded.

### 2.5. Criteria for Success

The filtering blebs were divided into 2 groups: a successful group and a failed group. Success was defined as eyes with IOP ≤ 18 mmHg and a >30% reduction in IOP at 1 year after surgery without antiglaucoma medications or additional surgical interventions. Failed blebs were defined when IOP was over 18 mmHg, or below 18 mmHg with antiglaucoma medications or if any additional surgical intervention was necessary. The definitions for success and failure were used in accordance with the Guidelines on Design and Reporting of Glaucoma Surgical Trials [14].

### 2.6. Surgical Technique

All trabeculectomies were performed by a single experienced glaucoma surgeon (T.Ż.) under peribulbar anesthesia. Dissection of a superior fornix-based conjunctival flap was made. Electrocautery was used to control episcleral bleeding. A rectangular (4 × 3 mm) scleral flap to a depth of half the scleral thickness was prepared and two small, 4 × 3 mm sponges with MMC (0.3 mg/mL) were placed under the conjunctiva and over the scleral flap for 3 min. The MMC was then rinsed out thoroughly with balanced salt solution. Excision of trabecular meshwork was performed with a Pierce punch and peripheral iridectomy was performed in all instances. The scleral flap was sutured with two releasable 10-0 Nylon sutures which were placed on both corners of the scleral flap and one fixed 10-0 Nylon suture at the center of the scleral flap. The conjunctiva was closed with 10-0 Nylon sutures. The standard postoperative regimen consisted of topical levofloxacin 5 times a day (for 10 days), 1% atropine 2 times a day (for 2 weeks) and dexamethasone 5 times a day in tapering doses over 10 weeks. Postoperative maneuvers such as bleb massage, removal of releasable sutures or laser suture lysis were performed when considered necessary by the attending ophthalmologist.

### 2.7. Morphological Analysis of the Vascularity of the Filtering Bleb

**Figure 1:**
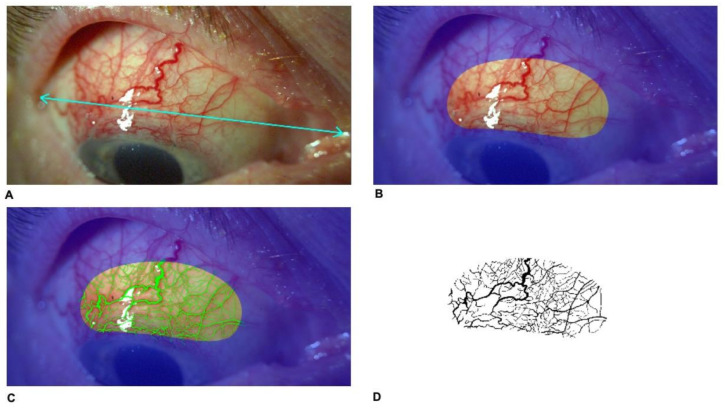
Slit-lamp anterior-segment photography of a filtering bleb 1 day after trabeculectomy. (**A**) Setting reference points (eye corners) on each standard photography in 10× magnification (blue arrow). (**B**) Creation of a constant bean-shaped area of 4.994 cm^2^ including scleral flap. (**C**) Adjusting the brightness of an image, increasing the contrast in RGB (red–green–blue) color model and enhancement of the green channel. The threshold was manually adjusted until the entire red area of vessels was highlighted in green. (**D**) After color correction of the photographs, processed masks were recorded as monochromatic (black and white) images and then black areas from the image were measured using ImageJ-aided quantification of black pixels to calculate the total vessel area.

In postoperative period, on day 1 and 14, 1, 3 and 6 months and 1 year postoperatively, slit-lamp photography in 10× magnification was performed in down gaze to obtain the maximal exposure of the bleb using Topcon SL-D701 with DC-4 photography system (Topcon Corporation, Tokyo, Japan). Original image size was 43.6 cm (width) and 32.7 cm (height), stored at 300 dpi in Tagged Image File Format (TIFF) format. Reference points, i.e., eye corners were determined (Figure 1A). Subsequently, a constant bean-shaped region of interest (ROI) including the scleral flap was identified (Figure 1B). Using Adobe Photoshop CS2 software (Adobe Systems Incorporated, San Jose, CA, USA), a file for each patient was created (Figure 1C). Determination of the bleb vessel area was performed by converting the image into three grey-scale images by using the red–green–blue stack function and then analyzing through the green channel where the contrast is greatest between red vessels and background. The threshold tool was used to segment the grey-scale image into the areas of interest and to exclude background. The threshold of the image was adjusted to best cover the vessel area. After color correction of the photographs, processed masks were recorded as monochromatic (black and white) images in TIFF and then put into the ImageJ program (imagej.net; version 1.49) (Figure 1D). The thresholded image is binary and will only show the vessel area region. Black areas from the image were measured using ImageJ-aided automatic quantification of black pixels to calculate the total vessel area. Then, the ratio of the bleb vessel area to the entire surface area of ROI was calculated and defined as vascularity of the bleb.

### 2.8. Statistical Analysis

Statistical analysis was performed using GraphPad Prism version 5.03 (GraphPad Software, San Diego, CA, USA). For continuous variables between groups, the independent Student’s *t*-test and the Mann–Whitney *U*-test were used for parametric and non-parametric analysis, respectively. Normality of data was assessed using Shapiro–Wilk test. Categorical variables were analyzed using the chi-square (χ^2^) test. For comparison of the vascularity of the filtering bleb (%) and IOP after the surgery, univariate linear regression analysis and Pearson’s correlation analysis were performed. Differences were considered statistically significant at *p* < 0.05.

## 3. Results

### 3.1. Patient Characteristics

Fifty-one eyes of 44 patients underwent MMC-augmented trabeculectomy in this study. All patients completed the follow-up schedule of 12 months and were analyzed. Study subjects comprised 25 males and 19 females and the mean age was 63.9 ± 10.5 years. There were 47 eyes with POAG and 4 eyes with PEXG. The mean preoperative IOP was 22.92 ± 4.70 mmHg. The mean postoperative IOP was 12.24 ± 3.92 mmHg on day 1 after surgery, 10.31 ± 4.55 mmHg after 1 week, 9.53 ± 3.57 mmHg after 2 weeks, 11.29 ± 3.98 mmHg after 1 month, 10.18 ± 3.77 mmHg after 3 months, 11.51 ± 3.66 mmHg after 6 months and 10.82 ± 3.69 mmHg after 1 year. The mean preoperative BCVA in LogMAR 1 day before surgery was 0.32 ± 0.34. One patient required bleb needling 9 months after surgery.

### 3.2. Rates of Surgical Success

At 1 year postoperatively, 40 eyes (78.4%) were classified as successful and 11 eyes (21.6%) as failed. Table 1 summarizes the demographics of the subjects in each group. There were no significant differences in terms of age, sex, type of glaucoma, preoperative IOP, BCVA and number of antiglaucoma medications.

### 3.3. Bleb Vascularity during the First 12 Months after Trabeculectomy

**Figure 2:**
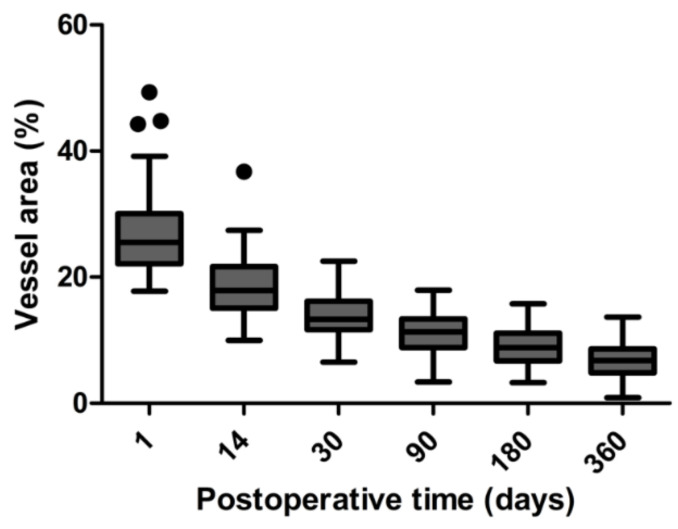
Box plot presentation of time-dependent changes of vascularity of the filtering blebs at all follow-up visits in the study. The change rate of the blood vessel area significantly decreased. Median values (horizontal lines), 25/75 percentiles (boxes), 5/95 percentiles (whiskers) and outliers (dots).

The peak in bleb vascularity was at day 1 after the surgery. In all patients, there was a statistically significant decrease in the blood vessel area over time (the *p* test for trend (*p* trend) was <0.0001). Figure 2 shows the vascularity of the filtering blebs during the first 12 months after trabeculectomy.

### 3.4. Bleb Vascularity and Surgical Success

Bleb vascularity at 1, 3 and 12 months after surgery was significantly higher in failed blebs (Table 2). Although not statistically significant, there was a trend in terms of higher conjunctival vessel density at all time points in the failure group. At 1 year after surgery, unsuccessful filtering blebs had significantly higher vessel density. The bleb vascularity in relation to the rates of success and failure at 12 months after surgery is presented in Table 2.

### 3.5. Bleb Vascularity and IOP

**Figure 3:**
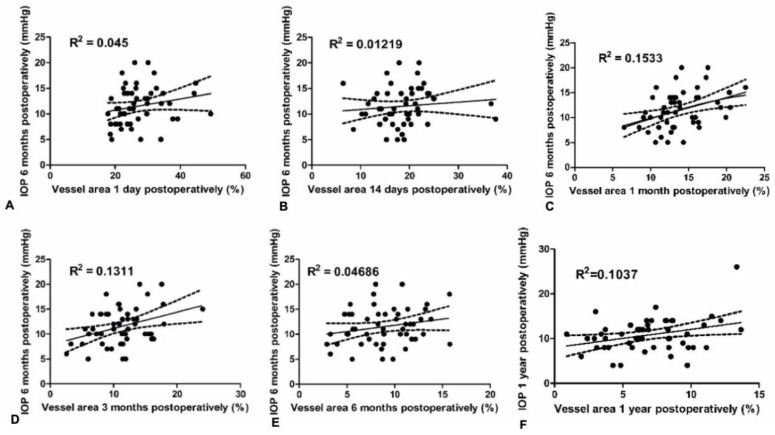
Scatterplots showing correlations between bleb vascularity (%) in early postoperative period and IOP 6 and 12 months after trabeculectomy. (**A**,**B**,**E**) There was no significant correlation between vascularity of the filtering bleb at day 1, 14 or 180 and IOP 6 months after trabeculectomy. (**C**,**D**) There was significant positive correlation between the vascularity of the filtering bleb 1 month postoperatively and IOP 6 months after surgery (*R*^2^ = 0.1533, *p* = 0.005) and 3 months postoperatively and IOP 6 months after surgery (*R*^2^ = 0.1311, *p* = 0.009). (**F**) There was significant positive correlation between bleb vascularity at 1 year and IOP 1 year after trabeculectomy (*R*^2^ = 0.1037, *p* = 0.0212).

The results of the linear regression analysis revealed a significant association between blood vessel area 1 month and 3 months after trabeculectomy, and IOP at 6 months postoperatively (F (1,49) = 8.873 and F (1,49) = 7.395, respectively) (Figure 3). Pearson’s correlation analysis confirmed the correlation between IOP at 6 months and bleb vascularity 1 month (*R*^2^ = 0.1533, *p* = 0.005) and 3 months postoperatively (*R*^2^ = 0.1311, *p* = 0.009). There was no significant correlation between vascularity of the filtering bleb at day 1, 14 or 180 with IOP 6 months after trabeculectomy (Figure 3). There was no significant correlation between vascularity of the filtering bleb at day 1, 14, 30, 90 or 180 and IOP 12 months after trabeculectomy. There was positive correlation between bleb vascularity at 1 year and IOP 1 year after trabeculectomy (Figure 3).

## 4. Discussion

Our study provides insight into the relationship between filtering bleb vascularization measured in an objective way on the color photography and the surgical outcomes after trabeculectomy. As the bleb vascularity plays an important role in postoperative wound healing after trabeculectomy, increased vascularity with reduction in bleb height and area are usually associated with a worse outcome of the surgery [15]. Thus, the presence of bleb vascularization in early postoperative period may be a poor prognostic factor. In many studies, authors have described bleb appearance in terms of subjectively assessed bleb pattern in conjunction with bleb vascularity measured on slit lamp examination. In most commonly used bleb grading systems (IBAGS, MBGS), bleb vascularity represents an assessment of the surface and deep vessel visibility over the surgical site [12,13]. However, these assessment methods are subjective and rely on clinical judgement with risk of variability among observers. Recently, new technologies, e.g., AS-OCT and OCT-A have been engaged for more precise and objective evaluation of the internal structure of filtering bleb. Nevertheless, in the clinical practice, slit lamp examination is the most commonly employed method to determine the characteristics of filtering blebs in glaucoma patients.

Previous studies demonstrated that increased vascularity of the filtering bleb affects its IOP-lowering function. Caglar et al. [16] analyzed blebs with IVCM and also observed that filtering blebs associated with an unsatisfactory IOP showed an abnormal conjunctival vascularization and tortuous vessels. Interestingly, Meziani et al. [17], who graded vascularization from 0 (poor) to 2 (hypervascularization) using AS-OCT, found no significant differences between functioning and non-functioning blebs. However, they performed AS-OCT examinations from 1.25 to 108 months after surgery. Increased bleb vascularization is a risk factor for filtering bleb failure in the early postoperative period. The reason of impaired bleb function after this time is due to scarring and tissue remodeling which takes place at the end of the wound healing process [18]. Interestingly, Hayek et al. [19] demonstrated that low preoperative conjunctival vessel density on the site of the future filtering bleb was associated with a lower IOP at 6 months. They measured conjunctival vascularization in OCT-A using ImageJ software.

Whereas many studies have investigated the internal morphology of the filtering bleb using AS-OCT, UBM or IVCM, so far there was only one study which objectively measured the vascularity of blebs. Yin et al. [5] showed that increase in the vessel area 1 month after surgery measured with OCT-A, was positively correlated with IOP 6 months after trabeculectomy. This finding is consistent with that of our study. Interestingly, this study also revealed an association between bleb vascularity 1 month after trabeculectomy and IOP 6 months postoperatively, with lack of correlation between bleb vascularity in the early postoperative days. The possible explanation for this finding may lie in the wound healing process. During the second week after injury, granulation tissue matures and the amounts of collagen increases. The density of blood vessels diminishes as collagen accumulates [20]. After the increase in pro-angiogenic factors and growth of blood vessels in the wound, the period of vascular pruning occurs [21]. Research has showed that wounds that heal faster and with less scar formation exhibit reduced inflammation and capillary growth, and a more rapidly maturing capillary network [22]. Therefore, increased bleb vascularity in the first few days after surgery, which is associated with inflammatory hyperemia, may not have such importance to the outcome of the procedure as compared to the bleb vessel pattern at the tissue remodeling phase 2 to 4 weeks after the injury. These findings are consistent with the results of our study.

In our study, the peak in bleb vascularity was 1 day after surgery. In contrast, in the study of Hayek et al. [19], vascular density peak was observed 1 week after surgery. On the other hand, Yin et al. [5] found a peak in vessel density 1 month after trabeculectomy. These observations could be explained by the fact that both research groups used OCT-A to measure vessel density and the investigation depth was 2.0 mm. OCT-A is a new technology for non-invasive and rapid measurement of vessel density in the ocular anterior segment but has several limitations. OCT-A cannot differentiate between arterial/venous and lymphatic vessels. Moreover, conjunctival vascular density was much higher on OCT-A scans when compared to slit-lamp images [19].

The major limitations of our study include bias associated with image acquisition artifacts and then image analysis. Calculation of the vessel area by binarization and adjusting the threshold can be another source of error because some small vessels may disappear while artifacts can transform into vessels. However, each image was assessed individually and threshold was adjusted manually by an experienced technician to correct any errors resulting from image analysis. Another limitation of our study is the discrepancy between the follow-up when evaluating surgical success and IOP. However, it could result from the fact that during the follow-up, increased IOP was lowered with antiglaucoma medications or additional surgical interventions and IOP eventually remained low. Inclusion of both eyes in seven patients may be prone to bias associated with environmental, genetic or patient-specific factors. A further limitation of our study was the need to take measurements after exporting images on external software. However, this methodology was used to ensure the repeatability of measurements and objectivity in exploring the relationship between bleb vascularization and surgical outcomes after trabeculectomy. In the clinic, slit-lamp examination is the most commonly used method of evaluating bleb characteristics such as bleb vascularity. A better understanding of the time-frame of evolution of filtering bleb and wound healing would certainly help clinicians adjust their postoperative management of glaucoma patients.

In conclusion, in this prospective study, we demonstrated a strong relationship between filtering bleb vascularity and the surgical outcome one year after trabeculectomy. Using anterior segment photography, we quantitively assessed blood vessel area on the surface of the filtering bleb in a reproducible and precise manner. Interestingly, we found that the vascularity of the filtering bleb during early postoperative days was not correlated with IOP and rates of success of trabeculectomy at one year. However, there was a relationship between bleb vessel area of the filtering bleb 1 month after trabeculectomy with surgical outcomes. These results may be useful in the postoperative care of patients after trabeculectomy.

## Figures and Tables

**Table 1:** Patients characteristics before surgery.

Characteristic	Mean ± SD		*p* Value
	Success *n* = 40	Failure *n* = 11	
Age (y)	62.98 ± 11.23	67.45 ± 7.22	0.152 ^b^
Sex (male/female)	25/15	4/7	0.121 ^c^
Preoperative IOP (mmHg)	23.46 ± 4.94	21.17 ± 3.74	0.145 ^a^
Number of antiglaucoma medications	2.21 ± 1.24	2.17 ± 1.34	0.949 ^b^
Type of glaucoma (POAG/PEXG)	37/3	10/1	0.942 ^c^
BCVA (LogMAR)	0.34 ± 0.38	0.25 ± 0.16	0.835 ^b^

^a^ Independent Student’s *t*-test; ^b^ Mann–Whitney *U* test; ^c^ χ^2^ test; IOP = intraocular pressure; POAG = primary open angle glaucoma; PEXG = pseudoexfoliation glaucoma; BCVA = best corrected visual acuity; LogMAR = logarithm of the minimum angle of resolution.

**Table 2:** Vascularity of the filtering bleb after trabeculectomy in relation to surgical success rates 12 months postoperatively. Data presented as mean ± SD (95% CI).

Bleb Vascularity (%)	Success (*n* = 40)	Failure (*n* = 11)	*p* Value
1 day	25.97 ± 7.49 (23.57–28.36)	28.92 ± 6.80 (24.35–33.48)	0.106
14 days	17.85 ± 5.28 (16.16–19.54)	21.18 ± 6.56 (16.77–25.59)	0.106
1 month	13.01 ± 3.38 (11.93–14.09)	16.31 ± 3.53 (13.94–18.69)	0.005
3 months	10.15 ± 3.47 (9.04–11.26)	14.93 ± 4.08 (12.19–17.67)	0.001
6 months	8.50 ± 3.24 (7.46–9.53)	10.29 ± 2.90 (8.35–12.24)	0.173
12 months	6.37 ± 2.85 (5.46–7.28)	8.99 ± 2.45 (7.35–10.63)	0.011

Mann–Whitney *U* test.

## Abbreviations

OCT-A = optical coherence tomography angiography; UBM = ultrasound biomicroscopy; IVCM = in vivo confocal microscopy; AS-OCT = anterior segment optical coherence tomography; IBAGS = Indiana Bleb Appearance Grading Scale; MBGS = Moorfields Bleb Grading System; MMC = mitomycin C; POAG = primary open angle glaucoma; PEXG = pseudoexfoliation glaucoma; IOP = intraocular pressure; PACG = primary angle closure glaucoma; AMD = age-related macular degeneration; BCVA = best corrected visual acuity; LogMAR = logarithm of the minimum angle of resolution; ROI = region of interest; TIFF = tagged image file format; CI = confidence interval; SD = standard deviation; ANOVA = analysis of variance.
